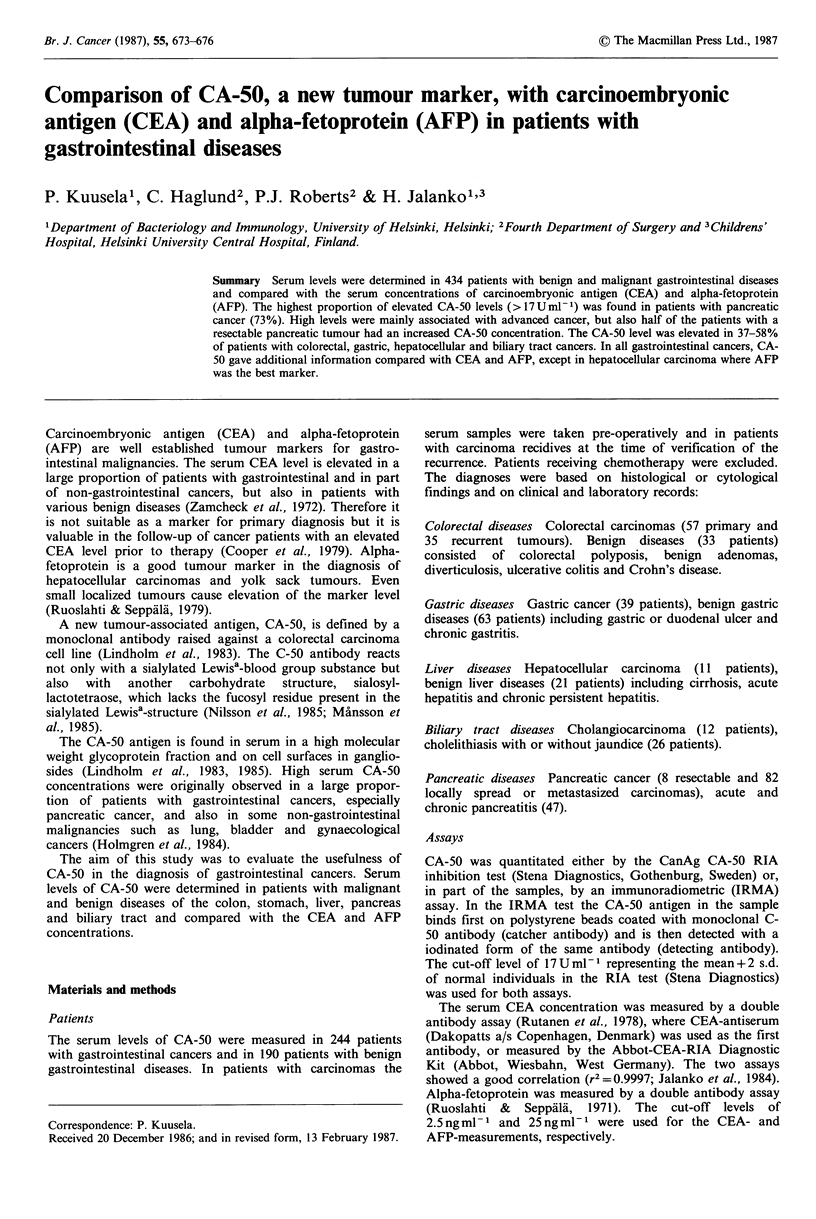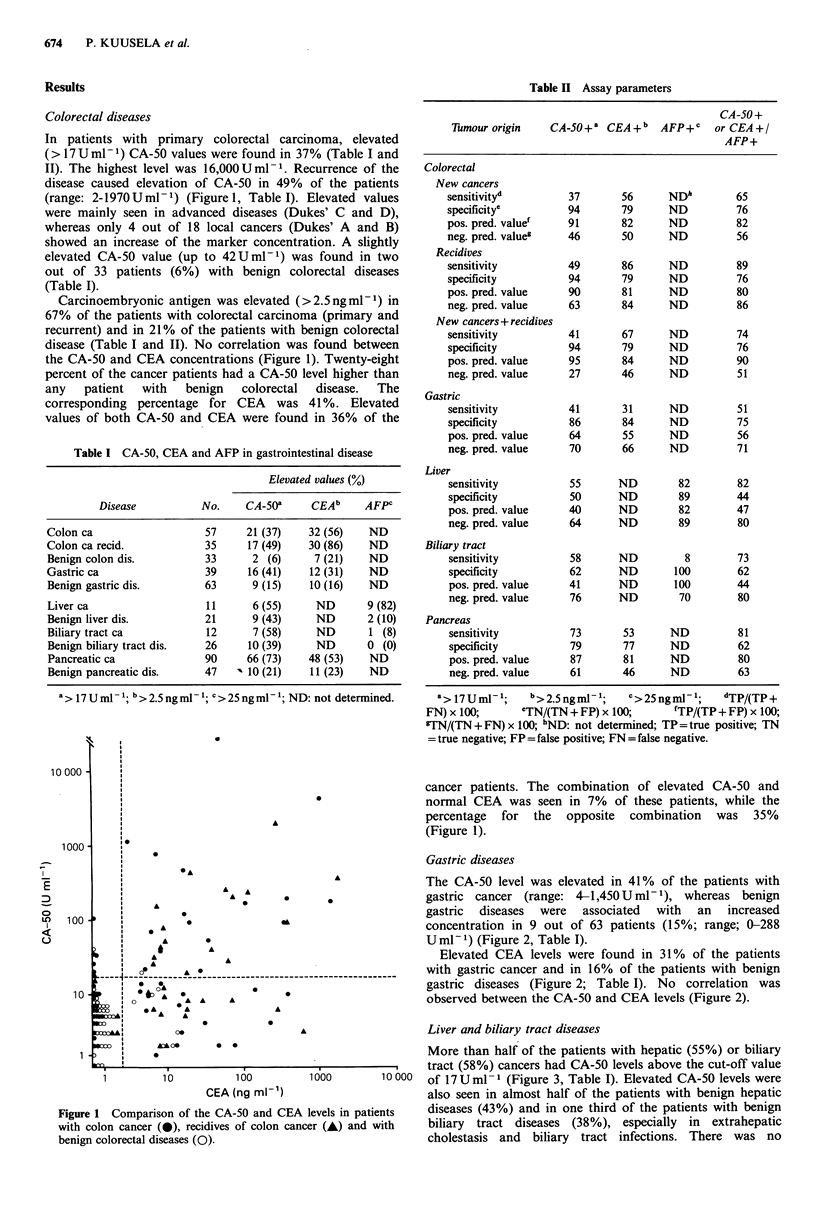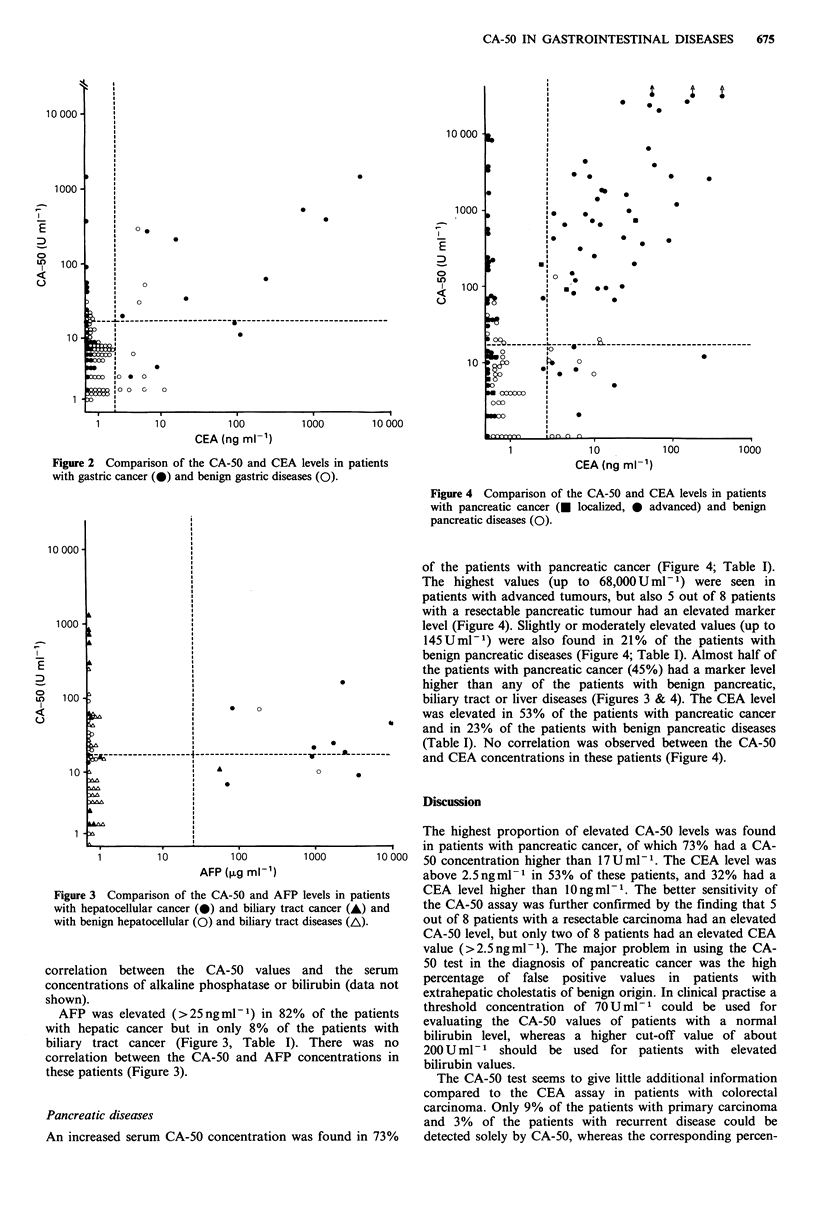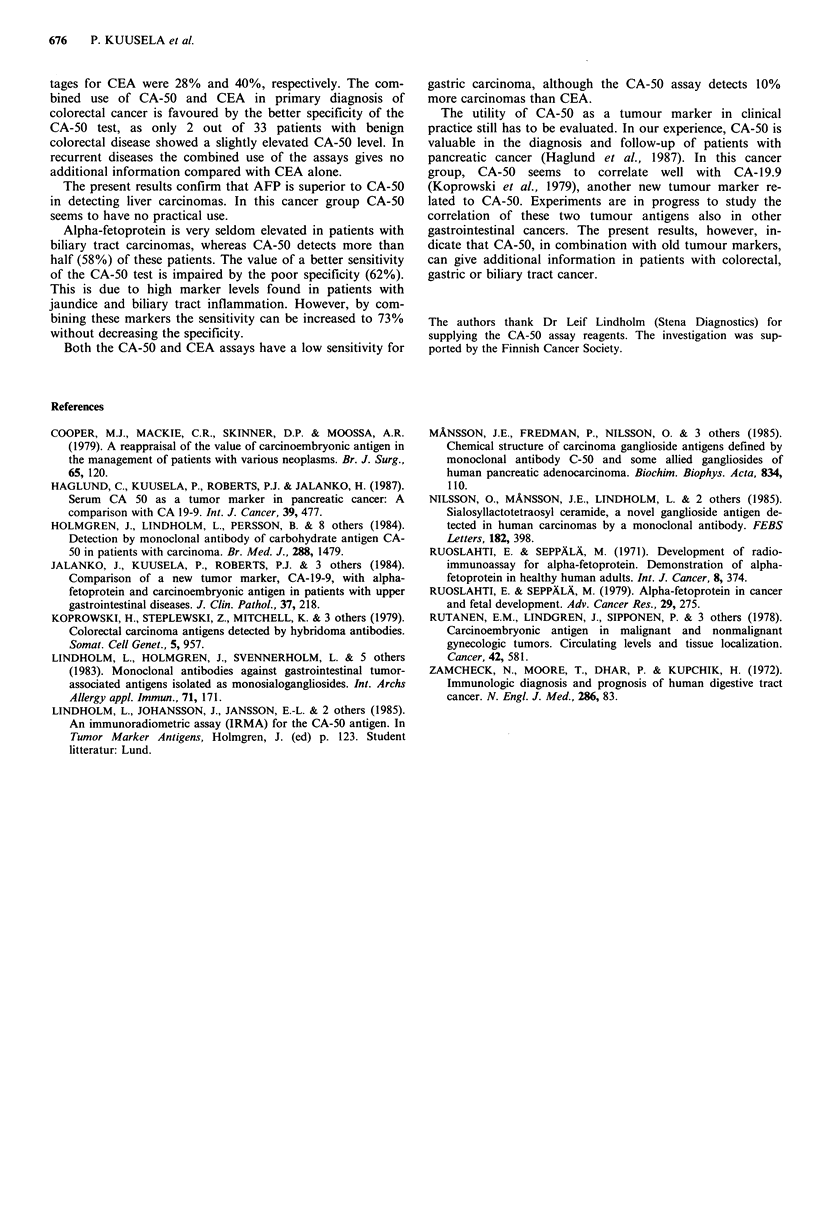# Comparison of CA-50, a new tumour marker, with carcinoembryonic antigen (CEA) and alpha-fetoprotein (AFP) in patients with gastrointestinal diseases.

**DOI:** 10.1038/bjc.1987.137

**Published:** 1987-06

**Authors:** P. Kuusela, C. Haglund, P. J. Roberts, H. Jalanko

## Abstract

Serum levels were determined in 434 patients with benign and malignant gastrointestinal diseases and compared with the serum concentrations of carcinoembryonic antigen (CEA) and alpha-fetoprotein (AFP). The highest proportion of elevated CA-50 levels (greater than 17 U ml-1) was found in patients with pancreatic cancer (73%). High levels were mainly associated with advanced cancer, but also half of the patients with a resectable pancreatic tumour had an increased CA-50 concentration. The CA-50 level was elevated in 37-58% of patients with colorectal, gastric, hepatocellular and biliary tract cancers. In all gastrointestinal cancers, CA-50 gave additional information compared with CEA and AFP, except in hepatocellular carcinoma where AFP was the best marker.


 © The Macmillan Press Ltd., 1987

### Comparison of CA-50, a new tumour marker, with carcinoembryonic antigen (CEA) and alpha-fetoprotein (AFP) in patients with gastrointestinal diseases

P. Kuusela[1], C. Haglund[2], P.J. Roberts[2] & H. Jalanko[1,3]

[1]*Department of Bacteriology and Immunology, University of Helsinki, Helsinki;* [2]*Fourth Department of Surgery and* [3]*Childrens' Hospital, Helsinki University Central Hospital, Finland.*

**Summary** Serum levels were determined in 434 patients with benign and malignant gastrointestinal diseases and compared with the serum concentrations of carcinoembryonic antigen (CEA) and alpha-fetoprotein (AFP). The highest proportion of elevated CA-50 levels ($>17\,\mathrm{U\,ml^{-1}}$) was found in patients with pancreatic cancer (73%). High levels were mainly associated with advanced cancer, but also half of the patients with a resectable pancreatic tumour had an increased CA-50 concentration. The CA-50 level was elevated in 37–58% of patients with colorectal, gastric, hepatocellular and biliary tract cancers. In all gastrointestinal cancers, CA-50 gave additional information compared with CEA and AFP, except in hepatocellular carcinoma where AFP was the best marker.

Carcinoembryonic antigen (CEA) and alpha-fetoprotein (AFP) are well established tumour markers for gastro-intestinal malignancies. The serum CEA level is elevated in a large proportion of patients with gastrointestinal and in part of non-gastrointestinal cancers, but also in patients with various benign diseases (Zamcheck *et al.*, 1972). Therefore it is not suitable as a marker for primary diagnosis but it is valuable in the follow-up of cancer patients with an elevated CEA level prior to therapy (Cooper *et al.*, 1979). Alpha-fetoprotein is a good tumour marker in the diagnosis of hepatocellular carcinomas and yolk sack tumours. Even small localized tumours cause elevation of the marker level (Ruoslahti & Seppälä, 1979).

A new tumour-associated antigen, CA-50, is defined by a monoclonal antibody raised against a colorectal carcinoma cell line (Lindholm *et al.*, 1983). The C-50 antibody reacts not only with a sialylated Lewis[a]-blood group substance but also with another carbohydrate structure, sialosyl-lactotetraose, which lacks the fucosyl residue present in the sialylated Lewis[a]-structure (Nilsson *et al.*, 1985; Månsson *et al.*, 1985).

The CA-50 antigen is found in serum in a high molecular weight glycoprotein fraction and on cell surfaces in gangliosides (Lindholm *et al.*, 1983, 1985). High serum CA-50 concentrations were originally observed in a large proportion of patients with gastrointestinal cancers, especially pancreatic cancer, and also in some non-gastrointestinal malignancies such as lung, bladder and gynaecological cancers (Holmgren *et al.*, 1984).

The aim of this study was to evaluate the usefulness of CA-50 in the diagnosis of gastrointestinal cancers. Serum levels of CA-50 were determined in patients with malignant and benign diseases of the colon, stomach, liver, pancreas and biliary tract and compared with the CEA and AFP concentrations.

### Materials and methods

### *Patients*

The serum levels of CA-50 were measured in 244 patients with gastrointestinal cancers and in 190 patients with benign gastrointestinal diseases. In patients with carcinomas the serum samples were taken pre-operatively and in patients with carcinoma recidives at the time of verification of the recurrence. Patients receiving chemotherapy were excluded. The diagnoses were based on histological or cytological findings and on clinical and laboratory records:

*Colorectal diseases* Colorectal carcinomas (57 primary and 35 recurrent tumours). Benign diseases (33 patients) consisted of colorectal polyposis, benign adenomas, diverticulosis, ulcerative colitis and Crohn's disease.

*Gastric diseases* Gastric cancer (39 patients), benign gastric diseases (63 patients) including gastric or duodenal ulcer and chronic gastritis.

*Liver diseases* Hepatocellular carcinoma (11 patients), benign liver diseases (21 patients) including cirrhosis, acute hepatitis and chronic persistent hepatitis.

*Biliary tract diseases* Cholangiocarcinoma (12 patients), cholelithiasis with or without jaundice (26 patients).

*Pancreatic diseases* Pancreatic cancer (8 resectable and 82 locally spread or metastasized carcinomas), acute and chronic pancreatitis (47).

### *Assays*

CA-50 was quantitated either by the CanAg CA-50 RIA inhibition test (Stena Diagnostics, Gothenburg, Sweden) or, in part of the samples, by an immunoradiometric (IRMA) assay. In the IRMA test the CA-50 antigen in the sample binds first on polystyrene beads coated with monoclonal C-50 antibody (catcher antibody) and is then detected with a iodinated form of the same antibody (detecting antibody). The cut-off level of $17\,\mathrm{U\,ml^{-1}}$ representing the mean + 2 s.d. of normal individuals in the RIA test (Stena Diagnostics) was used for both assays.

The serum CEA concentration was measured by a double antibody assay (Rutanen *et al.*, 1978), where CEA-antiserum (Dakopatts a/s Copenhagen, Denmark) was used as the first antibody, or measured by the Abbot-CEA-RIA Diagnostic Kit (Abbot, Wiesbahn, West Germany). The two assays showed a good correlation ($r^2 = 0.9997$; Jalanko *et al.*, 1984). Alpha-fetoprotein was measured by a double antibody assay (Ruoslahti & Seppälä, 1971). The cut-off levels of $2.5\,\mathrm{ng\,ml^{-1}}$ and $25\,\mathrm{ng\,ml^{-1}}$ were used for the CEA- and AFP-measurements, respectively.

Correspondence: P. Kuusela.
Received 20 December 1986; and in revised form, 13 February 1987.

### Results

### *Colorectal diseases*

In patients with primary colorectal carcinoma, elevated ($>17\,U\,ml^{-1}$) CA-50 values were found in 37% (Table I and II). The highest level was $16{,}000\,U\,ml^{-1}$. Recurrence of the disease caused elevation of CA-50 in 49% of the patients (range: $2\text{-}1970\,U\,ml^{-1}$) (Figure 1, Table I). Elevated values were mainly seen in advanced diseases (Dukes' C and D), whereas only 4 out of 18 local cancers (Dukes' A and B) showed an increase of the marker concentration. A slightly elevated CA-50 value (up to $42\,U\,ml^{-1}$) was found in two out of 33 patients (6%) with benign colorectal diseases (Table I).

Carcinoembryonic antigen was elevated ($>2.5\,ng\,ml^{-1}$) in 67% of the patients with colorectal carcinoma (primary and recurrent) and in 21% of the patients with benign colorectal disease (Table I and II). No correlation was found between the CA-50 and CEA concentrations (Figure 1). Twenty-eight percent of the cancer patients had a CA-50 level higher than any patient with benign colorectal disease. The corresponding percentage for CEA was 41%. Elevated values of both CA-50 and CEA were found in 36% of the cancer patients. The combination of elevated CA-50 and normal CEA was seen in 7% of these patients, while the percentage for the opposite combination was 35% (Figure 1).

**Table I** CA-50, CEA and AFP in gastrointestinal disease

| Disease | No. | Elevated values (%) CA-50[a] | CEA[b] | AFP[c] |
|---|---|---|---|---|
| Colon ca | 57 | 21 (37) | 32 (56) | ND |
| Colon ca recid. | 35 | 17 (49) | 30 (86) | ND |
| Benign colon dis. | 33 | 2 (6) | 7 (21) | ND |
| Gastric ca | 39 | 16 (41) | 12 (31) | ND |
| Benign gastric dis. | 63 | 9 (15) | 10 (16) | ND |
| Liver ca | 11 | 6 (55) | ND | 9 (82) |
| Benign liver dis. | 21 | 9 (43) | ND | 2 (10) |
| Biliary tract ca | 12 | 7 (58) | ND | 1 (8) |
| Benign biliary tract dis. | 26 | 10 (39) | ND | 0 (0) |
| Pancreatic ca | 90 | 66 (73) | 48 (53) | ND |
| Benign pancreatic dis. | 47 | 10 (21) | 11 (23) | ND |

[a]$>17\,U\,ml^{-1}$; [b]$>2.5\,ng\,ml^{-1}$; [c]$>25\,ng\,ml^{-1}$; ND: not determined.

**Figure 1** Comparison of the CA-50 and CEA levels in patients with colon cancer (●), recidives of colon cancer (▲) and with benign colorectal diseases (○).

**Table II** Assay parameters

| *Tumour origin* | *CA-50+*[a] | *CEA+*[b] | *AFP+*[c] | *CA-50+ or CEA+/ AFP+* |
|---|---|---|---|---|
| *Colorectal* | | | | |
| *New cancers* | | | | |
| sensitivity[d] | 37 | 56 | ND[h] | 65 |
| specificity[e] | 94 | 79 | ND | 76 |
| pos. pred. value[f] | 91 | 82 | ND | 82 |
| neg. pred. value[g] | 46 | 50 | ND | 56 |
| *Recidives* | | | | |
| sensitivity | 49 | 86 | ND | 89 |
| specificity | 94 | 79 | ND | 76 |
| pos. pred. value | 90 | 81 | ND | 80 |
| neg. pred. value | 63 | 84 | ND | 86 |
| *New cancers+recidives* | | | | |
| sensitivity | 41 | 67 | ND | 74 |
| specificity | 94 | 79 | ND | 76 |
| pos. pred. value | 95 | 84 | ND | 90 |
| neg. pred. value | 27 | 46 | ND | 51 |
| *Gastric* | | | | |
| sensitivity | 41 | 31 | ND | 51 |
| specificity | 86 | 84 | ND | 75 |
| pos. pred. value | 64 | 55 | ND | 56 |
| neg. pred. value | 70 | 66 | ND | 71 |
| *Liver* | | | | |
| sensitivity | 55 | ND | 82 | 82 |
| specificity | 50 | ND | 89 | 44 |
| pos. pred. value | 40 | ND | 82 | 47 |
| neg. pred. value | 64 | ND | 89 | 80 |
| *Biliary tract* | | | | |
| sensitivity | 58 | ND | 8 | 73 |
| specificity | 62 | ND | 100 | 62 |
| pos. pred. value | 41 | ND | 100 | 44 |
| neg. pred. value | 76 | ND | 70 | 80 |
| *Pancreas* | | | | |
| sensitivity | 73 | 53 | ND | 81 |
| specificity | 79 | 77 | ND | 62 |
| pos. pred. value | 87 | 81 | ND | 80 |
| neg. pred. value | 61 | 46 | ND | 63 |

[a]$>17\,U\,ml^{-1}$; [b]$>2.5\,ng\,ml^{-1}$; [c]$>25\,ng\,ml^{-1}$; [d]TP/(TP+FN) x 100; [e]TN/(TN+FP) x 100; [f]TP/(TP+FP) x 100; [g]TN/(TN+FN) x 100; [h]ND: not determined; TP = true positive; TN = true negative; FP = false positive; FN = false negative.

### *Gastric diseases*

The CA-50 level was elevated in 41% of the patients with gastric cancer (range: $4\text{–}1{,}450\,U\,ml^{-1}$), whereas benign gastric diseases were associated with an increased concentration in 9 out of 63 patients (15%; range; 0–288 $U\,ml^{-1}$) (Figure 2, Table I).

Elevated CEA levels were found in 31% of the patients with gastric cancer and in 16% of the patients with benign gastric diseases (Figure 2; Table I). No correlation was observed between the CA-50 and CEA levels (Figure 2).

### *Liver and biliary tract diseases*

More than half of the patients with hepatic (55%) or biliary tract (58%) cancers had CA-50 levels above the cut-off value of $17\,U\,ml^{-1}$ (Figure 3, Table I). Elevated CA-50 levels were also seen in almost half of the patients with benign hepatic diseases (43%) and in one third of the patients with benign biliary tract diseases (38%), especially in extrahepatic cholestasis and biliary tract infections. There was no

**Figure 2** Comparison of the CA-50 and CEA levels in patients with gastric cancer (●) and benign gastric diseases (○).

**Figure 3** Comparison of the CA-50 and AFP levels in patients with hepatocellular cancer (●) and biliary tract cancer (▲) and with benign hepatocellular (○) and biliary tract diseases (△).

correlation between the CA-50 values and the serum concentrations of alkaline phosphatase or bilirubin (data not shown).

AFP was elevated (>25 ng ml$^{-1}$) in 82% of the patients with hepatic cancer but in only 8% of the patients with biliary tract cancer (Figure 3, Table I). There was no correlation between the CA-50 and AFP concentrations in these patients (Figure 3).

*Pancreatic diseases*

An increased serum CA-50 concentration was found in 73%

**Figure 4** Comparison of the CA-50 and CEA levels in patients with pancreatic cancer (■ localized, ● advanced) and benign pancreatic diseases (○).

of the patients with pancreatic cancer (Figure 4; Table I). The highest values (up to 68,000 U ml$^{-1}$) were seen in patients with advanced tumours, but also 5 out of 8 patients with a resectable pancreatic tumour had an elevated marker level (Figure 4). Slightly or moderately elevated values (up to 145 U ml$^{-1}$) were also found in 21% of the patients with benign pancreatic diseases (Figure 4; Table I). Almost half of the patients with pancreatic cancer (45%) had a marker level higher than any of the patients with benign pancreatic, biliary tract or liver diseases (Figures 3 & 4). The CEA level was elevated in 53% of the patients with pancreatic cancer and in 23% of the patients with benign pancreatic diseases (Table I). No correlation was observed between the CA-50 and CEA concentrations in these patients (Figure 4).

### Discussion

The highest proportion of elevated CA-50 levels was found in patients with pancreatic cancer, of which 73% had a CA-50 concentration higher than 17 U ml$^{-1}$. The CEA level was above 2.5 ng ml$^{-1}$ in 53% of these patients, and 32% had a CEA level higher than 10 ng ml$^{-1}$. The better sensitivity of the CA-50 assay was further confirmed by the finding that 5 out of 8 patients with a resectable carcinoma had an elevated CA-50 level, but only two of 8 patients had an elevated CEA value (>2.5 ng ml$^{-1}$). The major problem in using the CA-50 test in the diagnosis of pancreatic cancer was the high percentage of false positive values in patients with extrahepatic cholestatis of benign origin. In clinical practise a threshold concentration of 70 U ml$^{-1}$ could be used for evaluating the CA-50 values of patients with a normal bilirubin level, whereas a higher cut-off value of about 200 U ml$^{-1}$ should be used for patients with elevated bilirubin values.

The CA-50 test seems to give little additional information compared to the CEA assay in patients with colorectal carcinoma. Only 9% of the patients with primary carcinoma and 3% of the patients with recurrent disease could be detected solely by CA-50, whereas the corresponding percentages for CEA were 28% and 40%, respectively. The combined use of CA-50 and CEA in primary diagnosis of colorectal cancer is favoured by the better specificity of the CA-50 test, as only 2 out of 33 patients with benign colorectal disease showed a slightly elevated CA-50 level. In recurrent diseases the combined use of the assays gives no additional information compared with CEA alone.

The present results confirm that AFP is superior to CA-50 in detecting liver carcinomas. In this cancer group CA-50 seems to have no practical use.

Alpha-fetoprotein is very seldom elevated in patients with biliary tract carcinomas, whereas CA-50 detects more than half (58%) of these patients. The value of a better sensitivity of the CA-50 test is impaired by the poor specificity (62%). This is due to high marker levels found in patients with jaundice and biliary tract inflammation. However, by combining these markers the sensitivity can be increased to 73% without decreasing the specificity.

Both the CA-50 and CEA assays have a low sensitivity for gastric carcinoma, although the CA-50 assay detects 10% more carcinomas than CEA.

The utility of CA-50 as a tumour marker in clinical practice still has to be evaluated. In our experience, CA-50 is valuable in the diagnosis and follow-up of patients with pancreatic cancer (Haglund *et al.*, 1987). In this cancer group, CA-50 seems to correlate well with CA-19.9 (Koprowski *et al.*, 1979), another new tumour marker related to CA-50. Experiments are in progress to study the correlation of these two tumour antigens also in other gastrointestinal cancers. The present results, however, indicate that CA-50, in combination with old tumour markers, can give additional information in patients with colorectal, gastric or biliary tract cancer.

The authors thank Dr Leif Lindholm (Stena Diagnostics) for supplying the CA-50 assay reagents. The investigation was supported by the Finnish Cancer Society.

### References

COOPER, M.J., MACKIE, C.R., SKINNER, D.P. & MOOSSA, A.R. (1979). A reappraisal of the value of carcinoembryonic antigen in the management of patients with various neoplasms. *Br. J. Surg.*, **65**, 120.

HAGLUND, C., KUUSELA, P., ROBERTS, P.J. & JALANKO, H. (1987). Serum CA 50 as a tumor marker in pancreatic cancer: A comparison with CA 19-9. *Int. J. Cancer*, **39**, 477.

HOLMGREN, J., LINDHOLM, L., PERSSON, B. & 8 others (1984). Detection by monoclonal antibody of carbohydrate antigen CA-50 in patients with carcinoma. *Br. Med. J.*, **288**, 1479.

JALANKO, J., KUUSELA, P., ROBERTS, P.J. & 3 others (1984). Comparison of a new tumor marker, CA-19-9, with alpha-fetoprotein and carcinoembryonic antigen in patients with upper gastrointestinal diseases. *J. Clin. Pathol.*, **37**, 218.

KOPROWSKI, H., STEPLEWSKI, Z., MITCHELL, K. & 3 others (1979). Colorectal carcinoma antigens detected by hybridoma antibodies. *Somat. Cell Genet.*, **5**, 957.

LINDHOLM, L., HOLMGREN, J., SVENNERHOLM, L. & 5 others (1983). Monoclonal antibodies against gastrointestinal tumor-associated antigens isolated as monosialogangliosides. *Int. Archs Allergy appl. Immun.*, **71**, 171.

LINDHOLM, L., JOHANSSON, J., JANSSON, E.-L. & 2 others (1985). An immunoradiometric assay (IRMA) for the CA-50 antigen. In *Tumor Marker Antigens*, Holmgren, J. (ed) p. 123. Student litteratur: Lund.

MÅNSSON, J.E., FREDMAN, P., NILSSON, O. & 3 others (1985). Chemical structure of carcinoma ganglioside antigens defined by monoclonal antibody C-50 and some allied gangliosides of human pancreatic adenocarcinoma. *Biochim. Biophys. Acta*, **834**, 110.

NILSSON, O., MÅNSSON, J.E., LINDHOLM, L. & 2 others (1985). Sialosyllactotetraosyl ceramide, a novel ganglioside antigen detected in human carcinomas by a monoclonal antibody. *FEBS Letters*, **182**, 398.

RUOSLAHTI, E. & SEPPÄLÄ, M. (1971). Development of radioimmunoassay for alpha-fetoprotein. Demonstration of alpha-fetoprotein in healthy human adults. *Int. J. Cancer*, **8**, 374.

RUOSLAHTI, E. & SEPPÄLÄ, M. (1979). Alpha-fetoprotein in cancer and fetal development. *Adv. Cancer Res.*, **29**, 275.

RUTANEN, E.M., LINDGREN, J., SIPPONEN, P. & 3 others (1978). Carcinoembryonic antigen in malignant and nonmalignant gynecologic tumors. Circulating levels and tissue localization. *Cancer*, **42**, 581.

ZAMCHECK, N., MOORE, T., DHAR, P. & KUPCHIK, H. (1972). Immunologic diagnosis and prognosis of human digestive tract cancer. *N. Engl. J. Med.*, **286**, 83.